
JOURNAL INFORMATION
==============================
NLM Title Abbreviation: Schizophr Bull
Journal ID: Schizophr Bull
NLM Journal ID: 0236760
Journal ID: schbul

Schizophrenia Bulletin

ISSN: 0586-7614
EISSN: 1745-1701
Publisher: Oxford University Press

ARTICLE INFORMATION
==============================
PMCID: PMC5887921
DOI: 10.1093/schbul/sby016.314
Article ID: sby016.314
Article version: 1
Subjects: Abstracts, Poster Session I

T38. Submission Withdrawn

Print publication date: 2018 Apr
Electronic publication date: 2018 Apr 1
Volume: 44
Issue: Suppl 1
Issue title: Abstracts for the Sixth Biennial SIRS Conference
First page: S127
Last page: S127
Copyright: © Maryland Psychiatric Research Center 2018.
Copyright year: 2018
License: This is an Open Access article distributed under the terms of the Creative Commons Attribution License (http://creativecommons.org/licenses/by/4.0/), which permits unrestricted reuse, distribution, and reproduction in any medium, provided the original work is properly cited.
License URL: https://creativecommons.org/licenses/by/4.0/

Page count: 1

==============================
### T38. SUBMISSION WITHDRAWN

### T39. NEURAL MECHANISMS OF METABOTROPIC GLUTAMATE RECEPTOR 3 MEDIATED ENHANCEMENT OF SYNAPTIC PLASTICITY AND COGNITION

Branden Stansley*[,1], Max Joffe[1], Samantha Yohn[1], Craig Lindsley[1], Colleen Niswender[1], P. Jeffrey Conn[2]
